# Long-range ordered vorticity patterns in living tissue induced by cell division

**DOI:** 10.1038/ncomms6720

**Published:** 2014-12-08

**Authors:** Ninna S. Rossen, Jens M. Tarp, Joachim Mathiesen, Mogens H. Jensen, Lene B. Oddershede

**Affiliations:** 1 Niels Bohr Institute, University of Copenhagen, Blegdamsvej 17, DK-2100 Copenhagen, Denmark

## Abstract

In healthy blood vessels with a laminar blood flow, the endothelial cell division rate is low, only sufficient to replace apoptotic cells. The division rate significantly increases during embryonic development and under halted or turbulent flow. Cells in barrier tissue are connected and their motility is highly correlated. Here we investigate the long-range dynamics induced by cell division in an endothelial monolayer under non-flow conditions, mimicking the conditions during vessel formation or around blood clots. Cell divisions induce long-range, well-ordered vortex patterns extending several cell diameters away from the division site, in spite of the system’s low Reynolds number. Our experimental results are reproduced by a hydrodynamic continuum model simulating division as a local pressure increase corresponding to a local tension decrease. Such long-range physical communication may be crucial for embryonic development and for healing tissue, for instance around blood clots.

## Abstract (web summary)

Endothelial cell division is highly sensitive to fluid conditions and essential for blood vessel healing. Here Rossen *et al.* demonstrate how cell division triggers the emergence of long-range votex patterns in endothelial tissue under conditions that mimic blood vessel formation and blood clots.

## Graphical abstract

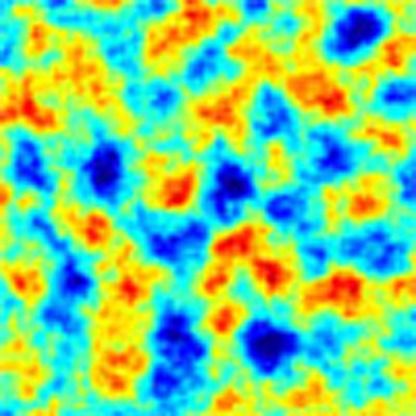

Endothelial cells line the blood vessels of the circulatory system—they are highly sensitive to fluid shear gradients^1^,^2^ and proliferate significantly more under no-stress conditions^3^. Cells in endothelial tissue adhere tightly to their neighbours to prevent leakage, thus causing the cells to move highly collectively^4^,^5^,^6^,^7^,^8^,^9^,^10^. Because of this tight adhesion to neighbouring cells, mechanical forces are transmitted over large distances across the tissue^11^. Such a mechanical signal can serve as a guide for the cells as they most often prefer to migrate in the direction of least shear stress^12^ and seem to navigate towards empty spaces^13^. A mechanical signal transmitted across the tissue can cause the cells to deform^14^,^15^, move^21^, divide^17^ and probably even differentiation can be mechanically controlled^18^,^19^. The living tissue is distinctly different from other materials as it consists of self-propelling cells that have a metabolism and can divide. Here we focus on how a cell division, which can be viewed as a local injection of energy, influences monolayer dynamics. The process of endothelial cell division is essential for correct embryo development^20^, angiogenesis and vessel repair^21^, as well as for the growth of metastasis from malignant tissue^22^.

## Results

### Dynamics and structure following cell division

**Figure 1:**
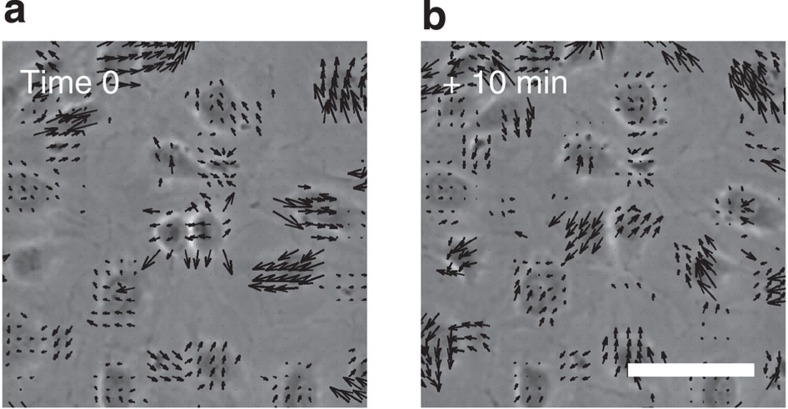
Velocity field around a cell division in endothelial monolayer. (**a**) First image taken after cytokinesis (time 0), the dividing cell is in the center of the image. (**b**) Image taken 10 min after cytokinesis at the same location as **a**. The vectors show the velocity field of the monolayer. The monolayer is confluent, all cells are connected with their nuclei (dark blobs) visually enhanced by phase contrast microscopy. Scale bar, 50 μm.

To examine the effect of cell division on flows in the endothelial monolayer, we tracked the motility of cells surrounding a division site. The division site was centred in the analysed frame, and the frame was rotated such that the daughter cells initially (time 0 in Fig. 1a) move apart in a horizontal direction. The monolayer had an average cell density of ~800 cells mm^−2^, a density that does not limit cell division^9^, and each cell had on average 6 neighbours as shown by Voronoi analysis (Supplementary Fig. 1). We used particle image velocimetry (PIV)^6^,^8^,^9^,^10^,^23^ to track the collective motion of cells every 10 min between 80 min before and 80 min after the central cell’s division. PIV analysis finds the maximum correlation between intensity patterns in two consecutive frames and returns the velocity field (shown as vectors in Fig. 1). We applied phase contrast microscopy, which facilitates the PIV analysis, particularly around cell nuclei. Time 0 is defined as the first image taken after cytokinesis where the cytoplasm of the mother cell is divided in two. Supplementary Figure 2 confirms that the PIV analysis correctly tracks the individual cells’ trajectories within the confluent monolayer.

**Figure 2:**
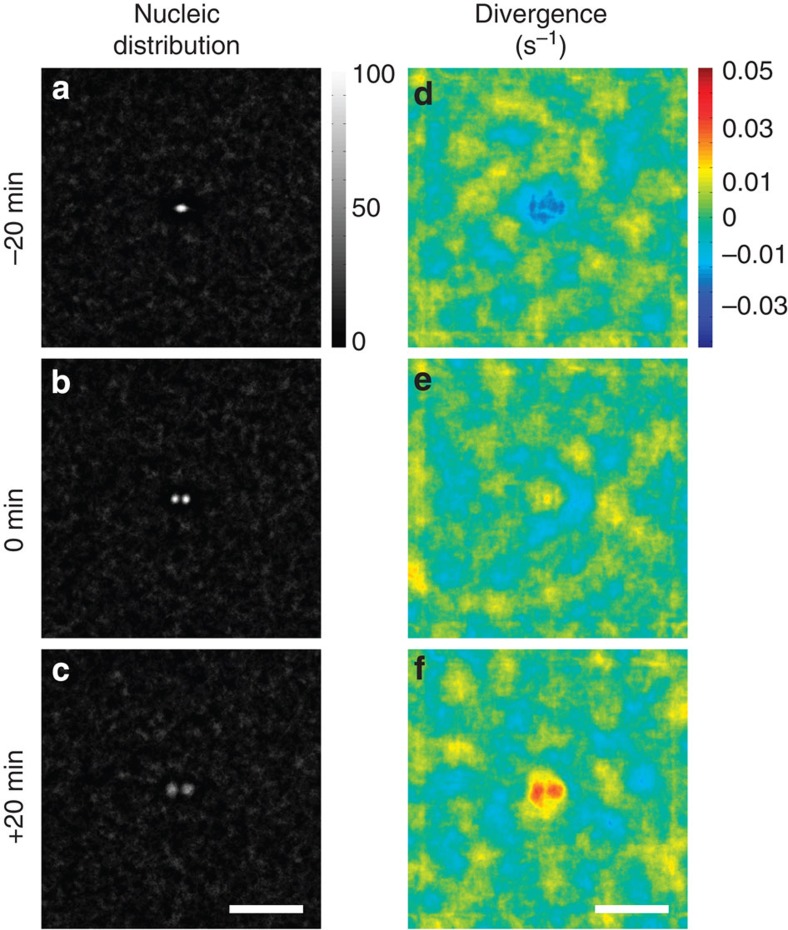
Average nuclei positions and divergence of the velocity field around a cell division. In the center of each frame, a cell divides and the daughter cells initially move in a horizontal direction, each image is an average of 100 samples. (**a**–**c**) Images derived from phase contrast microscopy (the nuclei appear bright and the cytoplasm dark) displaying the average positions of nuclei 20 min before (**a**), immediately after (**b**) and 20 min after (**c**) cytokinesis initiation. (**d**–**f**) Divergence of the velocity field at corresponding times before cytokinesis initiation (**d**), immediately after (**e**) and longer after (**f**) cytokinesis initiation. Scale bars, 80 μm.

To increase the signal-to-noise ratio in the analysis of the long-range velocity fields, we aligned and averaged over 100 cell divisions. Figure 2a–c shows the average nucleic positions of *n*=100 cell divisions (centred in the frame and rotated randomly, clockwise or anticlockwise, so that the dividing cells initially move in a horizontal direction) 20 min before (Fig. 2a), immediately after (Fig. 2b) and 20 min after (Fig. 2c) cytokinesis. During mitosis, the daughter nuclei move apart and in Fig. 1b a small counterclockwise rotation of the daughter cells with respect to the initial axis is visible, however, as detailed in Supplementary Fig. 3, the average rotation angle is (4.5±16.3°) which is not statistically significantly different from zero.

### Divergence and vorticity fields

The divergence field measures the net flow across a boundary region and is calculated using equation (4) from Methods. Figure 2d–f shows the average divergence field of 100 aligned cells 10 min before (Fig. 2d), immediately after (Fig. 2e) and 30 min after (Fig. 2f) cytokinesis. The divergence analysis proves that the tissue contracts towards the division site before cytokinesis and expands from the site of the the daughter cells after cytokinesis. Additional time frames of the divergence field is shown in Supplementary Fig. 4 together with the corresponding nucleic positions.

**Figure 3:**
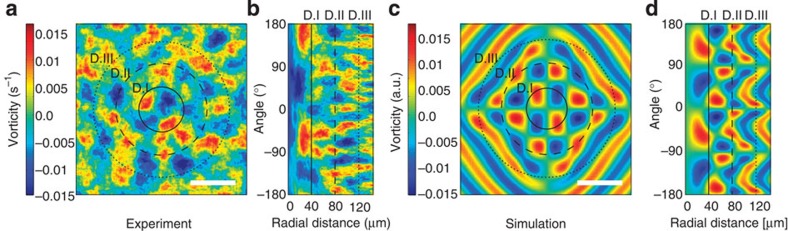
Long-range well-ordered vorticity pattern induced by cell division. The vorticity field emerging 30 min after cell division shown in cartesian coordinates (**a**) and polar coordinates (**b**). Images are the results of averaging 100 samples around an aligned cell division site. The full (D.I), dotted (D.II) and dashed (D.III) lines denote distances one, two and three cell diameters away from the division site, respectively, corresponding to radii of 40, 80 and 120 μm. (**c**,**d**) Vorticity field arising from a numerical simulation of the continuum model in cartesian coordinates (**c**) and in polar coordinates (**d**). Scale bars, 80 μm.

The vorticity field (calculated by equation (5) in Methods) describes the curl of the velocity field and carries important information about tissue flows induced by cell division. At a certain time, ~30 min after cell division, a distinct, long-range, well-ordered vortex pattern emerges as shown in cartesian (Fig. 3a) and polar coordinates (Fig. 3b), respectively. At the division site, there is no preferred sign of vorticity (as demonstrated in Supplementary Fig. 5). Adjacent to the division site two primary vortex pairs appear, with a clockwise (red) and a counterclockwise (blue) vortex flanking each daughter cell. These are located approximately one cell diameter away from the division site (D.I, full line in Fig. 3a,b). Well-ordered secondary and tertiary vortices are also induced by the cell division and appear farther from the division site: Approximately two cell diameters away (D.II, dashed lines in Fig. 3a,b), an ordered ring of eight vortex pairs is observed. Even at a distance of three cell diameters away from the division site (D.III, dotted lines in Fig. 3a,b) another ordered ring of vortices emerges, though at this distance from the central division center, the pattern is somewhat noisy due to the cell divisions taking place outside the framed region (no other cell divisions take place within the a distance of 6 cell diameters during the span of an experiment). A vortex is typically ~40 μm, as is the cell diameter. Supplementary Video 1 shows, in parallel, the time evolution of a dividing cell, the average nuclei positions and the accompanying divergence and vorticity fields.

**Figure 4:**
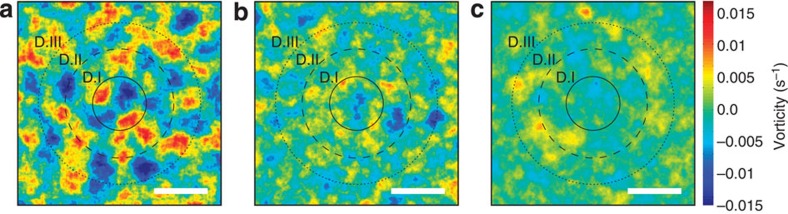
Extinction of the vorticity pattern over time. The average vorticity field shown for (**a**) 30 min (*n*=100), (**b**) 2 h and 30 min (*n*=71), (**c**) 4 h and 30 min (*n*=71) after cell division. During time, the long-range ordering becomes more blurred and finally goes extinct at a time long enough after the cell division. Scale bars, 80 μm.

To verify that our alignment procedure did not influence the results, several controls were made (two are shown in Supplementary Fig. 6) with alternative rotations of the data sets, these controls yield results consistent with Fig. 3. Even if a smaller subset of the data was used, *n*=30, a similar pattern occurred (Supplementary Fig. 7). Tissue areas without dividing cells or tissues where cell division had been chemically prohibited were also examined. These controls (shown in Supplementary Fig. 8) had significantly smaller vorticity fields and no long-range, ordered vorticity patterns. Hence, the pattern observed around a cell division is significantly different from random noise in the tissue. Also, monitoring the evolution of the vorticity over longer timescales, up to 4–5 h, as shown in Fig. 4 shows that the long-range ordered vorticity structures slowly lessen and disappear at times sufficiently long after cell division.

### Continuum model

To understand the physical origin of these long-range, well-ordered vorticity patterns arising from a cell division site in a two-dimensional (2D) tissue we formulated a continuum model, which was inspired by recent theoretical work reproducing the dynamics of bacterial suspensions^24^. The dynamics of the tissue is assumed to satisfy a momentum balance equation:


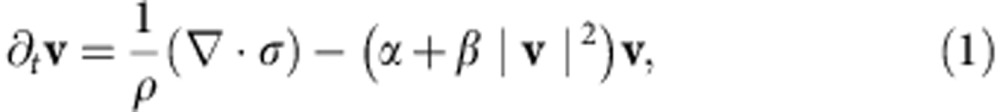


where *ρ* is the mean density, **v** the local mean velocity of the tissue and *σ* is the stress tensor. The latter two terms are parametrized by *α* and *β* and can be thought of as the positive half of a double well potential in |**v**|. Similar terms have also been employed to describe flocks and herds^25^. In this model, there is no inertial term, as the frictional forces totally dominate inertia for tissue movement. Stability requirements demand *β*>0, while *α* can have either sign. If *α*<0, in addition to the isotropic equilibrium state **v**=0, we get a non-trivial solution where cells move in an ordered state with a characteristic speed, 
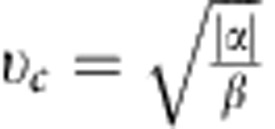
. Shortly after cell division, it is further assumed that the tissue moves as an incompressible fluid in which the projected area of each cell is conserved such that ∇·**v**=0. The stress tensor, *σ*, in equation (1) is assumed to have the form^24^:


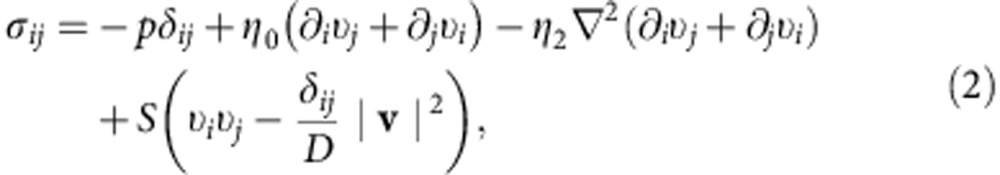


where *p* is the pressure. The linear momentum diffusion is parameterized by *η*_0_ and a higher-order dissipative term *η*_2_, a nematic term, similar to the Q-tensor for nematic crystals^26^, including a fitting parameter, *S*, and the spatial dimension of the setup, *D=2*, which describes the active stress contribution^24^. Comparing experimental data with the simulation, we find the fitted value *S*/*ρ*~2.1±0.2. In a tissue, the cells are self-propelling and continuously inject energy to move against viscous forces. In accordance with classical literature^27^,^28^,^29^ (and with the more recent ref. 24), we model this behaviour by setting *η*_0_<0. This negative viscosity, which is here introduced into our model, arises from the classical method of describing systems in which the fluctuation spectrum has a low non-zero extremal value and in which the viscosity can be renormalized through an expansion around the extremal point^27^,^28^,^29^. This expansion gives rise to an unstable second-order term and a higher-order stabilizing term (where the latter can be interpreted as a viscosity). In our experiments, a length scale of the order of a cell diameter sets the extremal point in the fluctuation spectrum, and we switch on the higher-order term *η*_2_>0 to ensure stability of the system. This causes the two uniform solutions to become unstable and form a dynamic state resembling tissue dynamics. Inserting the stress tensor into equation (1) yields the following model:


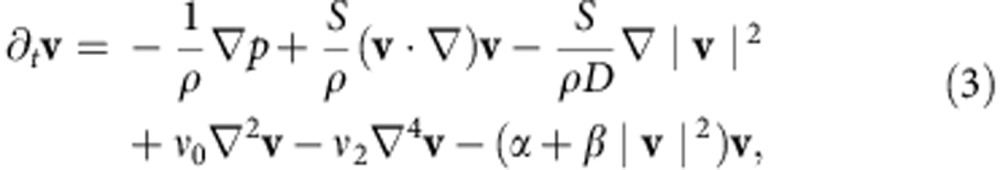


where *ν*_0_=*η*_0_/*ρ* and *ν*_2_=*η*_2_/*ρ*.

The symmetry is broken during cell division when the two daughter cells migrate in opposite directions. To model the cell division, we locally add a stokeslet dipole, *f*(*t*)(***δ***(**x**+**a**)−***δ***(**x**−**a**)), on the right-hand side of equation (3). The amplitude *f*(*t*) has the form of a square pulse in time and gives rise to two oppositely oriented forces (see details in Methods). The stokeslet dipole is aligned with the cell division axis and gives rise to a symmetry breaking. This pertubation locally and temporarily violates incompressibility; however, the system quickly relaxes and incompressibility is recovered.

One difference between our model and previous variants of the above equations^24^,^25^,^30^,^31^ is that our model considers the dynamics of a dense packing of cells, whereas previous works have focused on dilute suspensions of self-propelled particles. Our model is valid in a limit where the divergence of the elastic stress is balanced by the frictional forces between the cells and the substrate. Also, the velocities of interest here are approximately eight orders of magnitude slower than the elastic wave in the cytoplasm^32^. More details on the model, a stability analysis and explanation of the implementation of the numerical simulation of equation (3) are given in the Methods section.

The proposed model nicely captures the physics of the tissue dynamics and quantitatively reproduces the long-range vorticity pattern after cell division as shown in Fig. 3c. Also, the radial vorticity plot (Fig. 3d) is reproduced. The parameters entered into the model are the time of interest (30 min to compare with Fig. 3a,b) and the size of the analysed image (300 × 300 μm). A best fit of our model to the data returns an average speed of 1.4 μm min^−1^, which corresponds well to the experimentally observed average speed of 0.9 μm min^−1^. In addition, the velocity field, both the overall central patterns of the vector field and the absolute peak values, is well reproduced by the simulation (see Supplementary Fig. 9). This set of model parameters also returns the same divergence values as experimentally observed after cell division (Fig. 2).

### Fourier analysis

**Figure 5:**
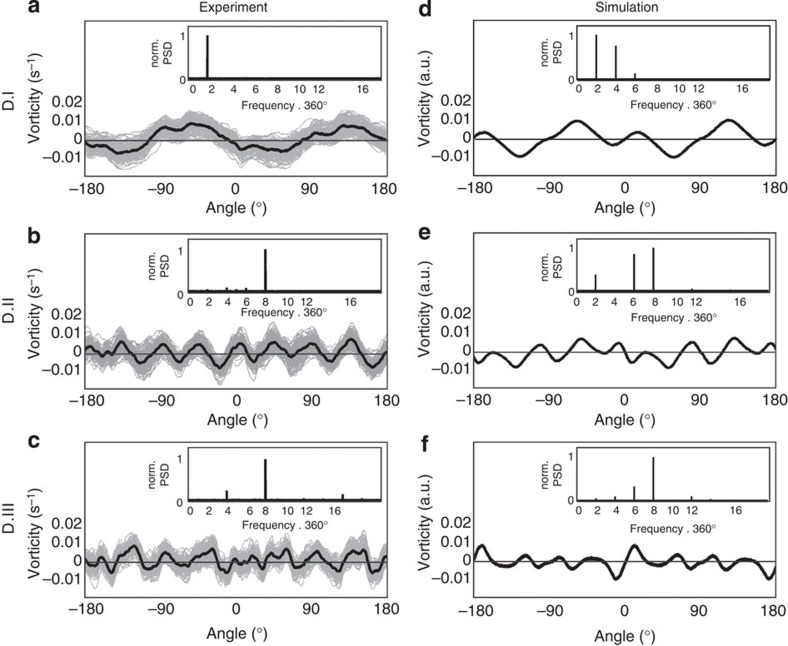
Quantification of primary, secondary and tertiary induced vortices. Vorticity as function of angle at radial distances of 1 (**a**), 2 (**b**) and 3 (**c**) cell diameters away from the cell division site 30 min after cell division. One hundred and twenty-nine different averages, each of 100 cells, are shown in light grey, their average is shown by a full black line. (**d**–**f**) the vorticity stemming from numerical simulation of the continuum model at the same distance from the division site as **a**–**c**, respectively. The insets show the corresponding power spectral densities.

To further quantify the induced long-range ordered vorticity pattern, we performed a Fourier analysis of the vorticity in bands at approximately one, two and three cell diameters away from the division site, these are shown in Fig. 5a–c. The grey lines in Fig. 5 show the vorticity as function of angle for 129 alternative rotations, the full black line shows their average. The simulated vorticity versus angle is shown in Fig. 5d–f. Both the experimental and the simulated vorticities have a periodic pattern and to determine the periodicity the corresponding power spectral densities were found (insets of Fig. 5). These quantify the number vortex pairs as two primary, eight secondary and eight tertiary vortex couples located one, two and three cell diameters away from the division site as visually apparent in Fig. 3a,b. Again, the numerical model reproduces the experimental data. Even if a smaller data set, *n*=30, is used, the same features are very clear in the power spectral analysis (Supplementary Fig. 10). Hence, the model captures the essential physics of tissue dynamics and this, combined with our experimental results, proves that the endothelial tissue displays hydrodynamic properties as a connected biomaterial rather than as a collection of individual cells.

## Discussion

The motility of cells in a confluent cellular monolayer is known to correlate over distances of ~200–350 μm (refs 6, 7), and correlated swirls exist over distances up to ~400 μm (ref. 6). In light of these distances, it is not surprising that the motion of new-born daughter cells stirs the endothelial monolayer and orders the tissue at distances of up to 140 μm away from the division site. As the daughter cells move apart in the endothelial monolayer, they exert a drag-force on the adhering neighbouring cells, thus creating two primary vortex couples. Such primary vortex couples have been reported for two cylinders moving apart in a continuous viscoelastic sheet^33^. However, the additional emergence of secondary and tertiary vortices in a predominantly viscous sheet has, to our knowledge, never been observed in a biological system before, but only in hydrodynamical turbulent systems characterized by high Reynolds numbers^34^. Using a characteristic speed of 1 μm min^−1^, a typical cell size of 40 μm (or a system size of 300 μm) and a cytoplasmatic viscosity of 14–17 poise (for human umbilical vein endothelial cells in the absence of vascular endothelial growth factor)^35^, we estimate a Reynolds number characterizing endothelial monolayer dynamics to be ~10^−9^; hence, tissue dynamics cannot be viewed as classical turbulence. However, as the tissue is not a Newtonian fluid, the dynamics is not well characterized by this Reynolds number.

Our continuum model that treats cell division as a local pressure increase, captures the tissue dynamics, both the velocity and vorticity fields. Tissue flows determine the orientation of the cells, and thereby of their division axes^17^. The orientation of the cell division axis could be decisive for whether a vessel lengthens or becomes thicker, hence, for the ability of an organism, for example, to counteract the clotting of a vessel, and this is a property that should be mimicked in artificial vessels. In literature, long-range tissue communications are attributed to chemical signalling^36^; however, our results show that the physical properties of tissue alone can explain long-range ordering and cellular communication. As stem cell differentiation can be mechanically regulated^37^ and the mechanisms governing morphogenesis are related to vortex formation 20, it may be that the hydrodynamical ordering following cell division also influences differentiation and morphogenesis.

## Methods

### Cell culture

Human umbilical vein endothelial cells (Invitrogen) were cultured in T25 flasks (Nunclon) with Endothelial Cell Basal Medium (Cell Applications). To create confluent monolayers, ~100,000 cells were seeded in Collagen IV-coated 30-mm circular dishes and cultured for 3 days at 37 °C and 5% CO_2_ with a media change every 24 h.

### Experiments

All experiments were conducted 3 days after seeding the cells. Phase contrast images (2,500 × 3,000 μm^2^) were taken of the monolayer every 10 min for 8 h. All dividing cells in the monolayer were located in the phase contrast images using a custom written MatLab programme that recognized the rounded cell before mitosis. Only isolated dividing cells, that is, cells that had no other dividing cell within a 240-μm radius for 20 min before to 30 min after, were used for this analysis. Over 1,000 dividing cells were identified and followed for 80 min before and after division, 100 of which could be considered ‘isolated’ using these restrictions.

### Image analysis

Image sequences of dividing cells were cropped from the larger phase contrast images. These images were aligned by centring the dividing cell in a 300 × 300 μm^2^ frame and rotating the frame so that the two daughter cells move away from the site of division along the horizontal axis immediately after mitosis (as shown in Fig. 1a). The rotation could in principle be done in two ways, either by rotating an angle *α* clockwise or an angle 180-*α* counterclockwise. We chose at random between the two and performed several controls where rotations were done differently (examples are shown in Supplementary Fig. 6).

### Particle image velocimetry

We used PIV to calculate the vector field describing the displacement of the cells’ nuclei between images taken 10 min apart. We used the PIVlab software package ( www.mathworks.com/matlabcentral/fileexchange/27659-pivlab-time-resolved-particle-image-velocimetry-piv-tool) for MATLAB (The MathWorks, Natick, MA) and used an interrogation area of 15.4 × 15.4 μm^2^ corresponding to 24 × 24 pixels^2^. The displacement and velocity vectors were calculated for each pixel.

### Divergence and vorticity

From the velocity vectors, we calculated the divergence and vorticity of the flow field. The divergence and vorticity were calculated for each pixel using the characteristic length scale of the system, the radius of the average cell area. If the cell density of a sample is 800 cells mm^−2^, then the cells have an average area of *A*=1,250 μm^2^, and the center of two neighbouring cells will be ~40 μm apart on average. The divergence, *d*, has SI units of (s^−1^) and measures the net flow (in units of (m^2^ s^−1^)), of the vector field across the smooth boundary of a small region, *A*, divided by its area in units of (m^2^). Hence, *d* was computed as:


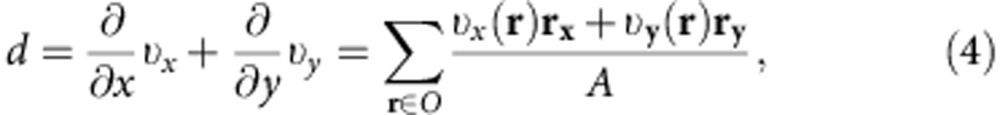


in which *O* is the circumference of the spherical area, *A*, 
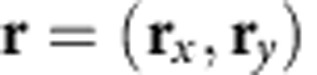
 is the vector from the center of *A* to a point on *O* and 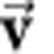 is the velocity vector on that point on *O*.

The strength of the vorticity, *ω*=(s^−1^), was computed as the amount of circulation, Γ=(m^2^ s^−1^), along the boundary of a small region, divided by the area of the small region:


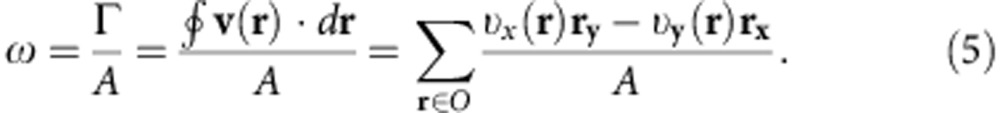


### More details on the continuum model

From ref. 32, we deducted the velocity of an elastic wave in the cytoplasm to be 1 m s^−1^. The length and timescales of interest for the current work are 10 μm over 10 min, hence, eight orders of magnitude slower than the elastic wave. Therefore, our model essentially followed from a balance between the divergence of the elastic stress and frictional forces, and not inertia. In the model, we assume the frictional forces to be proportional to the negative cell velocity and the stress to be proportional to the gradients in the displacement. Applying a time derivative to this balance gives an equation where the cell acceleration is proportional to the divergence of gradients in the velocity field. This essentially gives equation (1) where we in addition include contributions from the self-propelling force of the individual cells.

By taking the divergence of the vector field on both sides of equation (1), we achieve a modified version of the conventional Poisson equation for the pressure field encountered for incompressible Newtonian fluid flow,


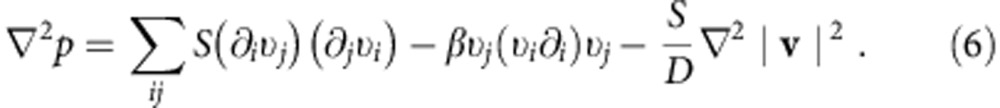


Experimentally, the dividing cell locally breaks the symmetry and chooses an axis along which the two daughter cells migrate in opposite directions. We model the cell division process as a stokeslet dipole, that is, by a local force perturbation along the *x* axis such that


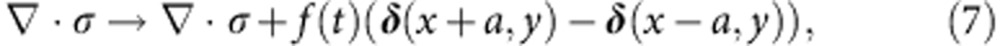


where we have introduced an amplitude *f*(*t*) of the local force and two *δ* functions. The amplitude of the force perturbation has the shape of a square pulse in time *f*(*t*)=*f*_0_ for 0≤*t*≤*t*_division_ otherwise *f*(*t*)=0, where we set *t*_division_=1 min. *f*_0_ is fitted such that the resulting amplitude of the velocity perturbation matches the experimentally measured amplitude. The scale *a* was in our simulation set to 7 μm in real units. In the simulation, the dipole is aligned with the axis of migration of the two new daughter cells.

By inserting the stress tensor, equation (2), in equation (1) we arrive at equation (3), which describes the tissue dynamics. Coupling equation (3) to the incompressibility equation, ∇·**v**=0, gives the continuum model used in the 2D simulation. Note that we need both the second- and fourth-order derivatives to describe the cell motion. Those two terms introduce a length scale comparable to the cell size, below which the dynamics is stable. That is, on the scale of individual cells, the dynamics is fairly coherent, whereas on larger scales the dynamics is controlled by unstable wavenumbers generating an inhomogeneous state of the velocity field. In the 2D case, this can be made further apparent by taking the curl of equation (3) linearized around the **v**=0 solution


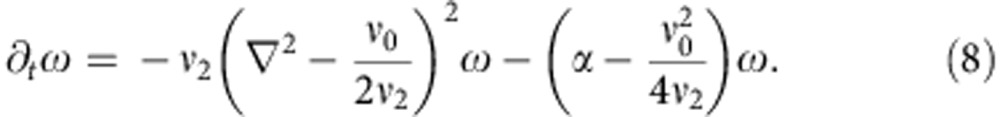


For *ν*_0_<0, this term will generate vorticity structures with an approximate wavelength of 
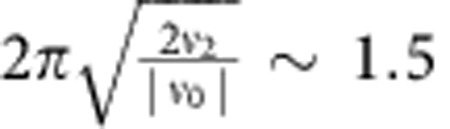
 cell diameters, which will describe a tendency for nearby cells to rotate in opposite directions. As can be seen from the stability analysis below, the long-range ordered state does not form when the second-order term is stabilizing the dynamics, that is, when *ν*_0_>0.

### Stability analysis

To examine further the dynamics of the model, we performed a linear stability analysis of equation (3) around a velocity field **v**_0_, which is independent of space and time^24^,^38^. Therefore, all derivatives in equation (3) vanish and the pressure computed from equation (6) is constant. Equation (3) then simplifies to


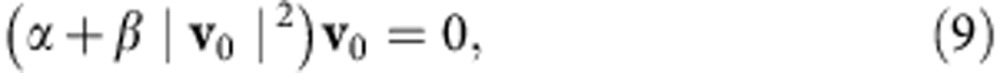


which has two solutions when *β*>0 and *α*<0, |**v**_0_|=0 and 
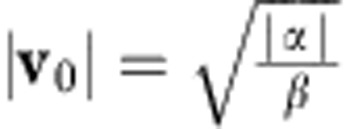
. We now perform a stability analysis of the these two solutions by perturbing both the velocity and the pressure fields by a small variation, **v**=**v**_0_+*δ***v** and *p*=*p*_0_+*δp*. To simplify the analysis, the coordinate system is rotated such that **v**_**0**_=(*v*_0_, 0) and *δ***v**=(ε_||_, ε_⊥_), with ε_||_, ε_⊥_ being the parallel and perpendicular components of the perturbation with respect to the velocity vector. Fourier transforming equation (3), we find to linear order in *δ***v**


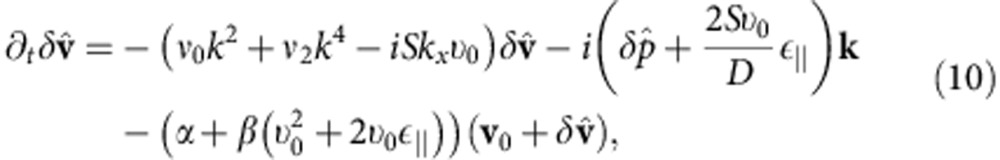


where the ‘^’ denotes transformed quantities and *k*^2^=*k*_*x*_^2^+*k*_*y*_^2^. For *υ*_0_=0, we find from the incompressibility condition, ∇·**v**=0, that the growth of the perturbation is on the form 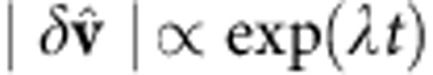, where *λ*=−(*α*+*ν*_0_*k*^2^+*ν*_2_*k*^4^). For *ν*_0_<0 and *ν*_2_>0, the uniform solution will be unstable to perturbations with wavenumbers in a narrow interval. For 
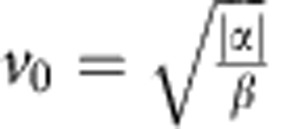
 the last term in equation (10) can be simplified into


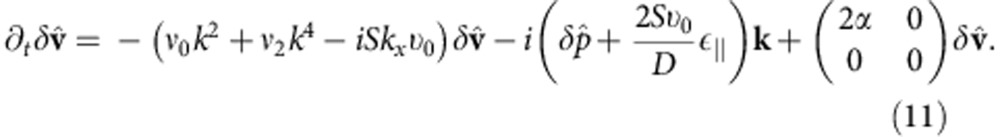


Again using the incompressibility condition, we find that


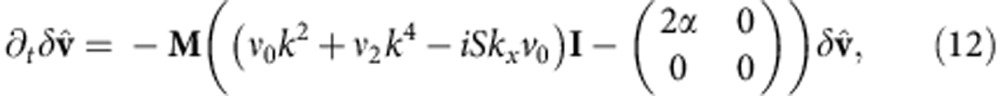


where **I** is the identity matrix and


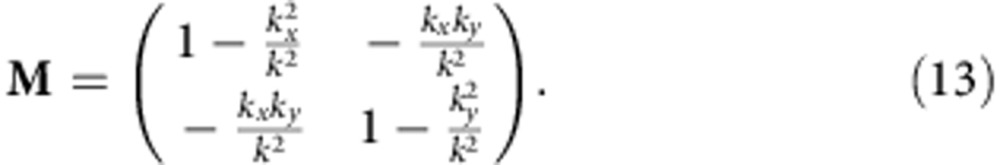


The eigenvalues for the non-zero solution are *λ*=0 and 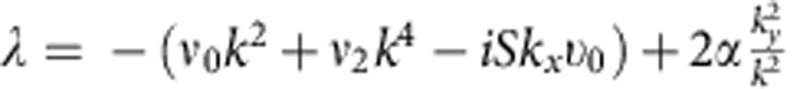. Once again for *ν*_0_<0 and *ν*_2_>0, we find a region of unstable wavenumbers making both fixed points simultaneously unstable. In Supplementary Fig. 11, we show the results of the stability analysis where the region of the unstable wavenumbers can be observed for the two fixed points, respectively. We further observe that for *α*<0 and *η*_0_<0, both fixed points will be unstable when perturbed by long wavelengths implying that equation (3) describes an inhomogeneous velocity field.

### Model parameter estimation

The five model parameters with a physical dimension are given by


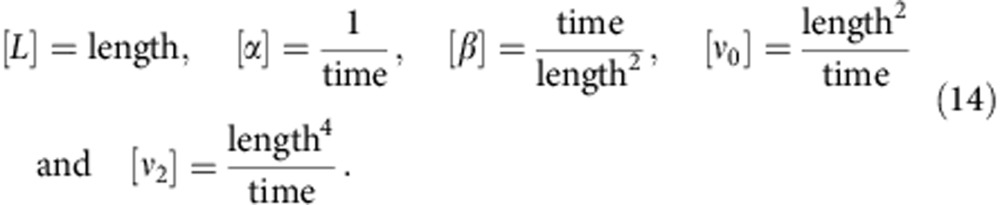


In general, *α* and *β* describe the properties of the isotropic states, while *ν*_0_ and *ν*_2_ describe the emergence and evolution of the vorticity patterns. Combining the dimensional quantities, we find that the velocity of the ordered state is given by 
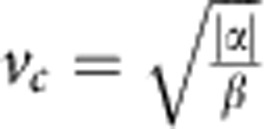
, which roughly corresponds to the crawling velocity of the cells.

The length scale *L* is set to size of the experimental system. The four remaining parameters have been fitted by minimizing the distance between the vorticity field of the model and the experiments in a circle with a radius of approximately three cell diameters and centred on a cell division event. To convert the dimensionless model parameters to real parameters with units, we use a timescale and the length scale *L*. In the experiment, the vorticity pattern develops fully over the course of ~*t*=30 min, which corresponds to *t*=11.4·*t*_s_ in simulation time. From that we estimate the time conversion factor from the simulation time to be *t*_s_=2.63 min. The physical parameters and their corresponding physical values and SI units are provided in Supplementary Table 1. The full simulation spans a 4*π* × 4*π* square. To ensure that the boundary effects from the simulation’s periodic boundaries are negligible, only the central square region of the simulation is used. The length conversion factor is found to be *l*_s_≈47.7 μm. Using the conversion factors, *t*_s_ and *l*_s_, and the values from Supplementary Table 1, we find the characteristic cell speed to be v_*c*_≈1.4 μm min^−1^ in good agreement with the experimental value (0.9 *μ*m min^−1^).

### Numerical simulation

The model was simulated in a 2D box with periodic boundary conditions using a pseudospectral method. After Fourier transforming equation (1), the resulting equations were solved numerically by an exponential time integration scheme^39^. The non-linear terms were evaluated in real space and then transformed back to Fourier space by repeated use of the Fast Fourier Transform and its inverse. To suppress aliasing errors, the 3/2-rule has been implemented^40^. The stability of the simulations were tested for a wide range of parameters and on grid sizes ranging from 128 × 128 to 512 × 512 with timesteps of the order Δ*t*~10^−4^. To ensure that the flow field remained incompressible, a pressure correction term was implemented effectively driving the divergence of the velocity field towards zero^40^. The simulation was initialized with a divergence-free flow field, which was allowed to relax. Thereafter, the perturbation in the pressure was inserted and the flow field relaxed once more, while the vorticity field was extracted.

## Author contributions

N.S.R. and L.B.O. designed the research. N.S.R. carried out the experiments and conducted the analysis. J.M. and M.H.J. proposed the model and J.M.T. carried out the simulation. N.S.R., J.M., J.M.T. and L.B.O. wrote the manuscript. M.H.J, J.M. and L.B.O. oversaw the project.

## Additional information

**How to cite this article**: Rossen, N. S. *et al.* Long-range ordered vorticity patterns in living tissue induced by cell division. *Nat. Commun.* 5:5720 doi: 10.1038/ncomms6720 (2014).

## Supplementary Material

Supplementary Information Supplementary Figures 1-11 and Supplementary Table 1

Supplementary video Long-range ordered vorticity patterns in living tissue induced by cell division
