# Supplementary material for: Long-range ordered vorticity patterns in living tissue induced by cell division
Source: Nat Commun. 2014 Dec 8;5:5720. doi: 10.1038/ncomms6720 (PMC4268690; doi:10.1038/ncomms6720)
Supplement: Supplementary Information — Supplementary Figures 1-11 and Supplementary Table 1 [file ncomms6720-s1.pdf]

Supplementary Material for Rossen *et al.*

Supplementary Figures

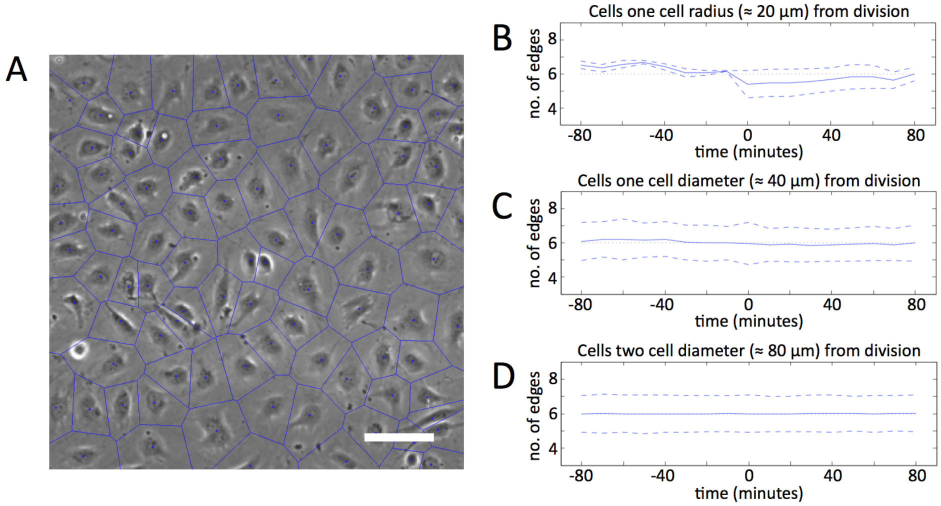

Supplementary Figure 1: **Voronoi analysis of cell packing in the monolayer**. A: Example of a Voronoi diagram super-positioned on an image of the dividing cells. The bar indicates 50 μm. B, C and D: The blue line indicates the average number of edges or neighbors for the cells at a half (B), one (C), and two (D) cell diameters away from the central cell division. Standard deviation is denoted with a dashed blue line. The typical number of edges is 6. Note a drop in the average number of edges from 6 to ~ 5 at the site (B) and time (t=0) of cell division. n=30.

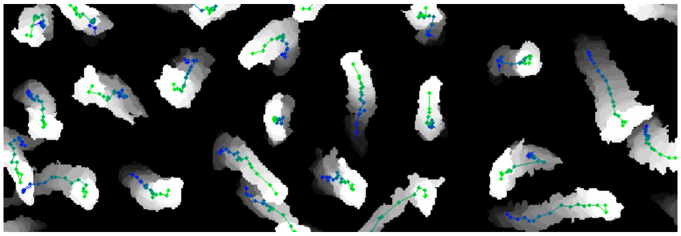

Supplementary Figure 2: **The trajectories predicted by PIV analysis are in accordance with the observed cell nuclei positions**. The endothelial cell nuclei are darker in phase contrast microscopy and can be found using image analysis. Here, the nuclei have been printed in grey and white, the dark grey denote the initial positions and white the final positions of the nuclei. The trajectories were predicted using PIV. The PIV predicted trajectories starts out blue and turns green at the final position.

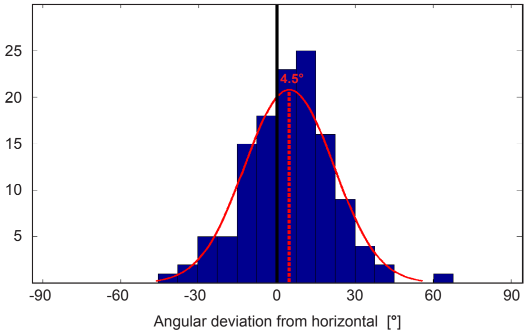

Supplementary Figure 3: **Histogram of the daughter cells' angular deviation from horizontal 20 minutes after cell division**. The average rotation of the division axis is 4.5° ± 16.3°, which is not statistically significantly different from 0, n=100.

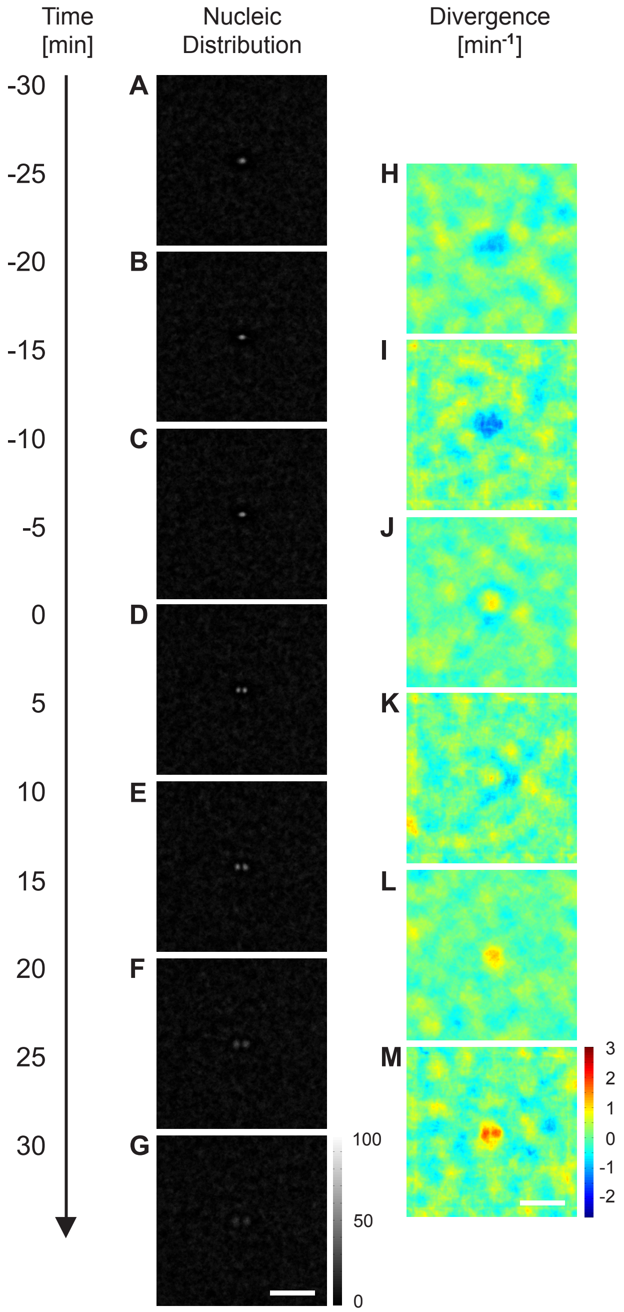

Supplementary Figure 4: **The average nucleic positions and divergence during cytokinesis**. The average nucleic position (A-G) and the average divergence of the vorticity fields (H-M) from 30 minutes before to 30 minutes after cell division taken over 100 datasets. We denoted the time immediately after division (cytokinesis) as time 0. The scale bars indicate 80 μm.

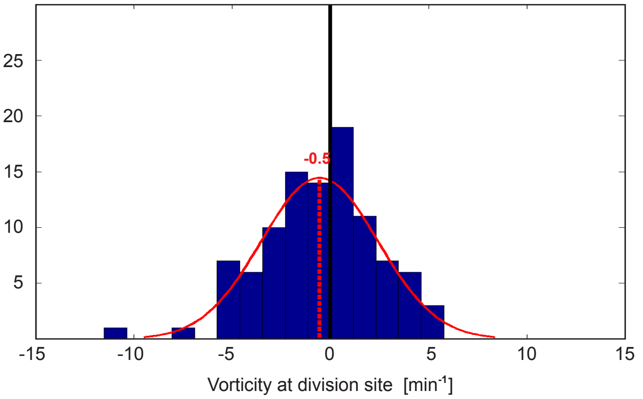

Supplementary Figure 5: **Histogram of vorticity at division site for 100 datasets between 20 and 30 minutes (i.e., 25 minutes) after cell division**. The average vorticity of the division axis is -0.54 min^{-1} $\pm$ 2.97 min^{-1}, which is not statistically significantly different from 0, n=100.

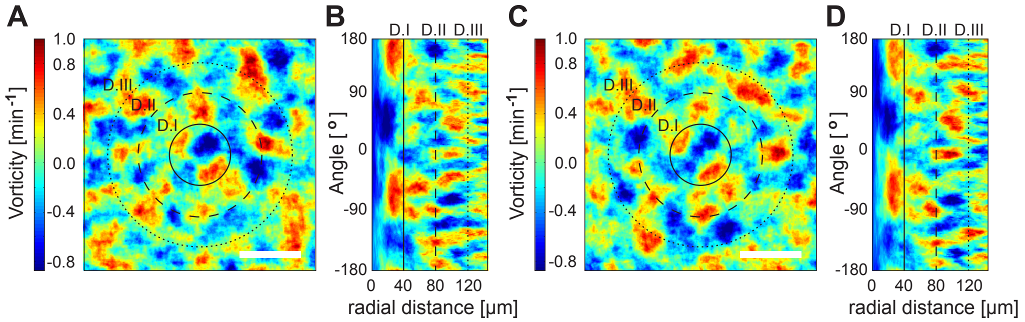

Supplementary Figure 6: **Alternative rotations yield similar long-range, well-ordered vorticity pattern**. The frames for each individual cell division can be rotated either clockwise or counterclockwise to align with the horizontal axis. (A) and (B) show two average vorticity field from different rotations of the 100 cell divisions than the rotations used to align the cell division samples in Figure 3. As in Figure 3, the full, dotted and dashed lines denote the approximate rows of neighboring cells one, two, and three cell diameters away from the division corresponding to the radii of 40, 80, and 120 μm. The scale bars are 80 μm.

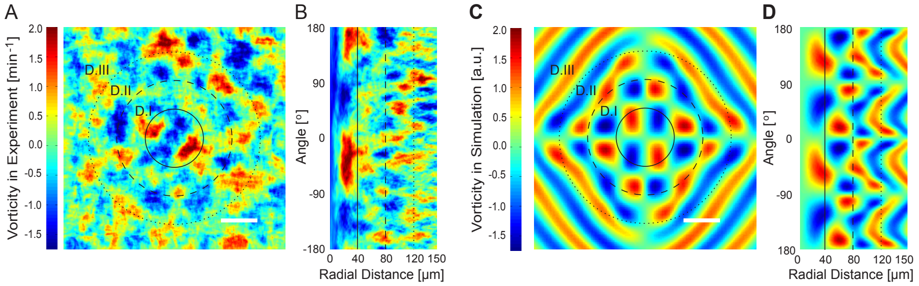

Supplementary Figure 7: **Vorticity 30 minutes after cell division for smaller dataset**. This figure is similar to Figure 3 of the manuscript, the only difference being that this figure is an average taken over only n=30 datasets, in the manuscript we used n=100. The emerging patterns are the same, regardless of whether 30 or 100 datasets were used in the average. The scale bars are 50 μm.

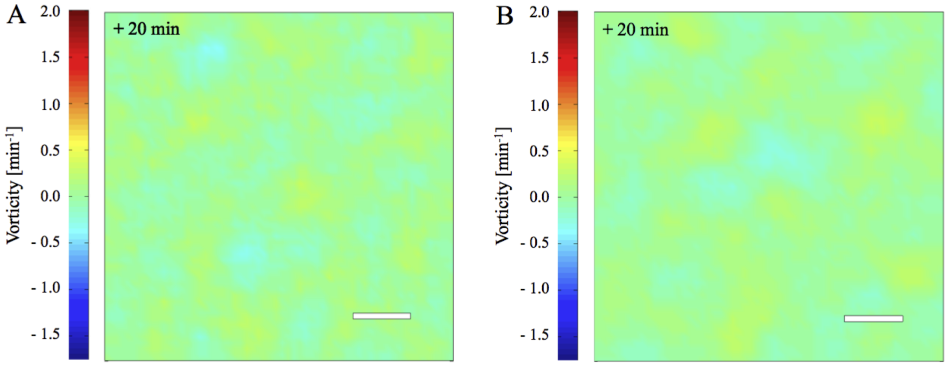

Supplementary Figure 8: **Control samples without divisions in the center do not yield a vorticity pattern**. The average vorticity field of 30 control samples in which the central cells were not dividing (A). The vorticity field of samples in which the cells were treated with aphidicolin and could not divide, n = 30 (B). The scale bars are 50 μm.

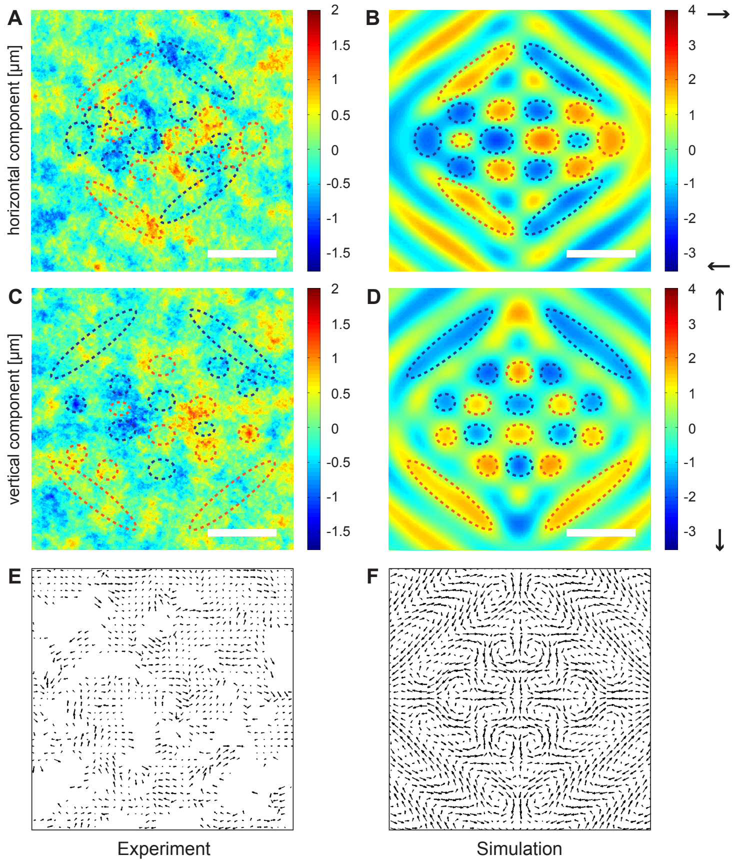

Supplementary Figure 9: **Experimental and simulated velocity fields**. A comparison of the experimentally found and simulated velocity fields. A)-D) show the vertical and horizontal components of the velocity fields from the experiments (left) and simulations (right) 30 minutes after cell division. A) and B) show the horizontal components of the velocity fields, C) and D) the vertical components, both given in the same units. E) shows the vector velocity field from a single experiment 30 minutes after cell division and F) the corresponding simulation (same scales as A)-D)). The PIV analysis follows gradients in the image, hence, in practice, PIV follows the location of the nuclei. The vacant areas in E) are caused by the lack of an intensity gradient (a nucleus) at those locations in that particular experiment. There is a reasonable agreement between experiments and simulations also of the velocity fields, especially considering the relatively large spread inherently present in the experimental data. The scale bars are 80 μm.

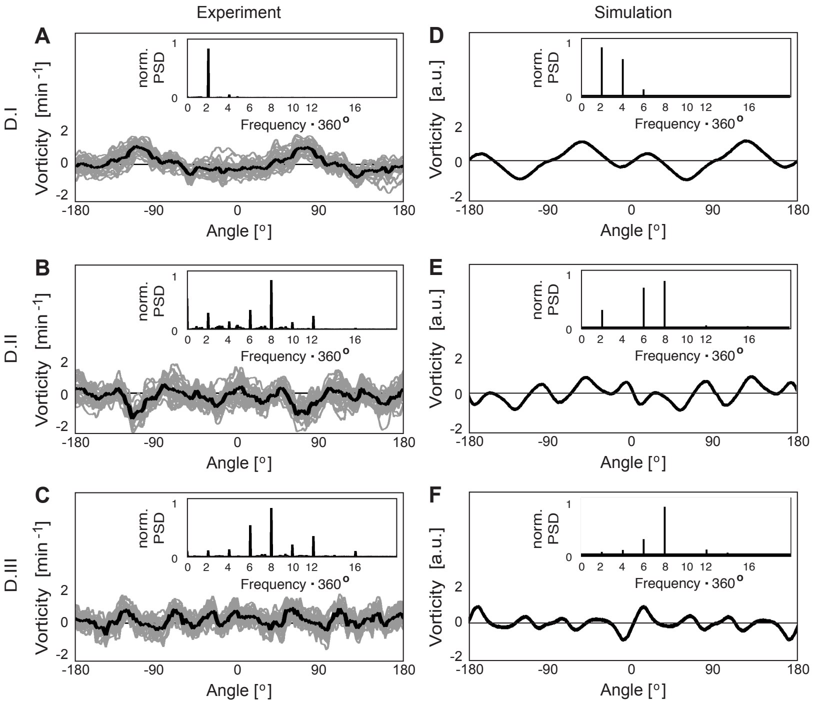

Supplementary Figure 10: **Power spectral analysis 30 minutes after cell division for smaller dataset**. This figure is similar to Figure 4 of the manuscript, the only difference being that this figure is an average taken over only n=30 datasets, in the manuscript we used n=100. The power spectra are essentially the same, regardless of whether 30 or 100 datasets were used in the average.

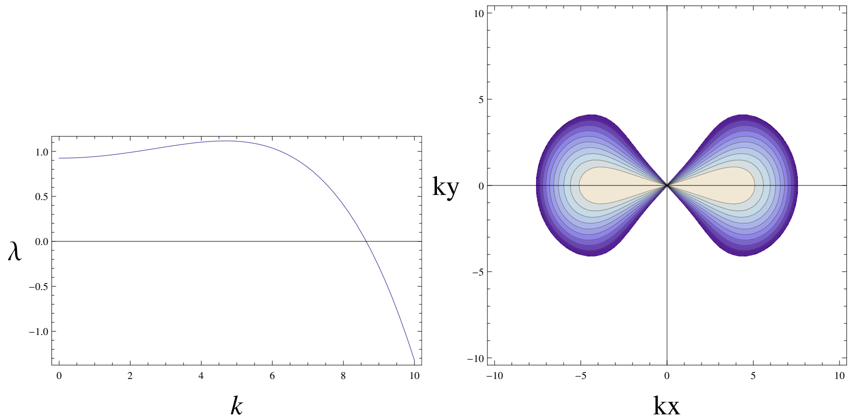

Supplementary Figure 11: **Region of unstable wave numbers for the two fixed points**. In the left panel, we show the result of the linear stability analysis around the fixed point $|\mathbf{v}_0| = 0$. For $\lambda > 0$, the fixed point is unstable. In the right panel, we show the similar stability analysis for the other fixed point $|\mathbf{v}_0| = \sqrt{\frac{|\alpha|}{\beta}}$, where the unstable wave numbers are located in the blue region. Note that we here have perturbed around a uniform flow, $\mathbf{v}_0$, pointing in the x-direction.

Supplementary Table

Parameters	Simulation values	Physical values		SI units	
α	-0.925	-0.352	min^{-1}	$-5.85 \cdot 10^{-3}$	s^{-1}
β	148	0.172	$\text{min}/\mu\text{m}^2$	$1.03 \cdot 10^{13}$	s/m^2
ν_0	-0.0175	-15.1	$\mu\text{m}^2/\text{min}$	$-2.52 \cdot 10^{-13}$	m^2/s
ν_2	0.0004	784	$\mu\text{m}^4/\text{min}$	$1.31 \cdot 10^{-23}$	m^4/s
η_0				$-2.52 \cdot 10^{-10}$	Pa·s
η_2				$1.31 \cdot 10^{-20}$	m·kg/s

Supplementary Table 1 The parameter values in the simulation and the physical values to which they correspond.
